# Regulatory reliance to approve new medicinal products in Latin American and Caribbean countries

**DOI:** 10.26633/RPSP.2021.10

**Published:** 2021-04-09

**Authors:** Carlos E. Durán, Martín Cañás, Martín A. Urtasun, Monique Elseviers, Tatiana Andia, Robert Vander Stichele, Thierry Christiaens

**Affiliations:** 1 Ghent University Ghent Belgium Ghent University, Ghent, Belgium.; 2 Federación Médica de la Provincia de Buenos Aires (FEMEBA) La Plata Argentina Federación Médica de la Provincia de Buenos Aires (FEMEBA), La Plata, Argentina.; 3 Universidad de los Andes Bogotá Colombia Universidad de los Andes, Bogotá, Colombia.

**Keywords:** Pharmaceutical preparations, government agencies, drug approval, United States Food and Drug Administration, Pan American Health Organization, Latin America, Caribbean region, Preparaciones farmacéuticas, agencias gubernamentales, aprobación de drogas, United States Food and Drug Administration, Organización Panamericana de la Salud, América Latina, región del Caribe, Preparações farmacêuticas, órgãos governamentais, aprovação de drogas, United States Food and Drug Administration, Organização Pan-Americana da Saúde, América Latina, região do Caribe, Applicable regulation to approve new medicinal products via regulatory reliance in Latin America and the Caribbean

## Abstract

**Objective.:**

To describe the current status of regulatory reliance in Latin America and the Caribbean (LAC) by assessing the countries’ regulatory frameworks to approve new medicines, and to ascertain, for each country, which foreign regulators are considered as trusted regulatory authorities to rely on.

**Methods.:**

Websites from LAC regulators were searched to identify the official regulations to approve new drugs. Data collection was carried out in December 2019 and completed in June 2020 for the Caribbean countries. Two independent teams collected information regarding direct recognition or abbreviated processes to approve new drugs and the *reference* (trusted) regulators defined as such by the corresponding national legislation.

**Results.:**

Regulatory documents regarding marketing authorization were found in 20 LAC regulators’ websites, covering 34 countries. Seven countries do not accept reliance on foreign regulators. Thirteen regulatory authorities (Argentina, Colombia, Costa Rica, Dominican Republic, Ecuador, El Salvador, Guatemala, Mexico, Panama, Paraguay, Peru, Uruguay, and the unique Caribbean Regulatory System for 15 Caribbean States) explicitly accept relying on marketing authorizations issued by the European Medicines Agency, United States Food and Drug Administration, and Health Canada. Ten countries rely also on marketing authorizations from Australia, Japan, and Switzerland. Argentina, Brazil, Chile, and Mexico are reference authorities for eight LAC regulators.

**Conclusions.:**

Regulatory reliance has become a common practice in the LAC region. Thirteen out of 20 regulators directly recognize or abbreviate the marketing authorization process in case of earlier approval by a regulator from another jurisdiction. The regulators most relied upon are the European Medicines Agency, United States Food and Drug Administration, and Health Canada.

## RESUMEN

**Objetivo.:**

Describir el estado actual de la utilización de las decisiones de autoridades regulatorias de otras jurisdicciones en América Latina y el Caribe mediante la evaluación de los marcos regulatorios nacionales para la aprobación de nuevos medicamentos y establecer los organismos regulatorios extranjeros que se consideran autoridades regulatorias confiables para cada país.

**Métodos.:**

Se realizaron búsquedas en los sitios web de las autoridades regulatorias de América Latina y el Caribe para identificar las regulaciones oficiales para la aprobación de nuevos medicamentos. La recopilación de datos se llevó a cabo en diciembre del 2019 y se completó en junio del 2020 para los países del Caribe. Dos equipos independientes recopilaron información sobre el reconocimiento directo o los procedimientos abreviados para la aprobación de nuevos medicamentos y los autoridades regulatorias *de referencia* (confiables) así definidos en la legislación nacional correspondiente.

**Resultados.:**

Se encontraron documentos regulatorios sobre la aprobación de nuevos productos en los sitios web de veinte organismos regulatorios de América Latina y el Caribe, que abarcaban 34 países. Siete países no aceptan la utilización de decisiones de autoridades regulatorias extranjeras. Trece autoridades regulatorias (Argentina, Colombia, Costa Rica, Ecuador, El Salvador, Guatemala, México, Panamá, Paraguay, Perú, República Dominicana, Uruguay y el sistema regulador único para quince Estados del Caribe) aceptan de manera explícita confiar las decisiones para aprobación de nuevos medicamentos emitidas por la Agencia Europea de Medicamentos, la Administración de Alimentos y Medicamentos de Estados Unidos y Salud Canadá. Diez países aceptan también utilizar las autorizaciones para la comercialización de Australia, Japón y Suiza. Argentina, Brasil, Chile y México son autoridades de referencia para ocho autoridades regulatorias en la región.

**Conclusiones.:**

La utilización de las decisiones de autoridades regulatorias de otras jurisdicciones se han convertido en una práctica común en América Latina y el Caribe. Trece de veinte autoridades regulatorias reconocen directamente o abrevian el proceso de aprobación de nuevos medicamentos en caso de que hayan recibido previamente la aprobación por parte de un organismo regulatorio de otra jurisdicción. La Agencia Europea de Medicamentos, la Administración de Alimentos y Medicamentos de Estados Unidos y Salud Canadá son las autoridades regulatorias de otras jurisdicciones en las cuales los reguladores de América Latina y el Caribe confían más.

## RESUMO

**Objetivo.:**

Descrever a prática atual de uso de decisões regulatórias de outras jurisdições na América Latina e no Caribe (ALC) mediante avaliação os marcos regulatórios dos países para aprovação de novos medicamentos e verificar, para cada país, quais entidades reguladoras estrangeiras são consideradas autoridades reguladoras de confiança por cada país.

**Métodos.:**

Foi realizada uma pesquisa nos sites das autoridades reguladoras da ALC para identificar as regulamentações oficiais para aprovação de novos medicamentos. A coleta de dados foi feita em dezembro de 2019 e concluída em junho de 2020 para os países do Caribe. Dois grupos independentes coletaram informações sobre o reconhecimento direto ou o procedimento abreviado para aprovação de novos medicamentos e as autoridades reguladoras de referência (de confiança) definidas como tal pela respectiva legislação nacional.

**Resultados.:**

Documentos regulatórios relacionados à aprovação de novos produtos foram obtidos de 20 sites de órgãos reguladores da ALC, abrangendo 34 países. Sete países não admitem o uso de decisões regulatórias de entidades reguladoras externas. Treze autoridades reguladoras (na Argentina, Colômbia, Costa Rica, El Salvador, Equador, Guatemala, México, Panamá, Paraguai, Peru, República Dominicana, Uruguai e o Sistema Regulador do Caribe unificado para 15 Estados caribenhos) admitem explicitamente a admissibilidade de decisões regulatórias para aprovação de novos medicamentos de outras jurisdições, quais sejam: Agência Europeia de Medicamentos (EMA), Agência Reguladora de Alimentos e Medicamentos (FDA) dos EUA e Health Canada. Dez países também aceitam decisões para autorização de comercialização da Austrália, Japão e Suíça. Argentina, Brasil, Chile e México são autoridades de referência para oito agências reguladoras.

**Conclusões.:**

O uso de decisões regulatórias de outras jurisdições tornou-se prática comum na América Latina e Caribe. Treze das 20 agências reguladoras reconhecem diretamente ou abreviam o procedimento de aprovação de novos medicamentos no caso de tal aprovação já haver sido concedida por uma autoridade reguladora de outra jurisdição. A EMA, a FDA e a Health Canada são as autoridades estrangeiras nas quais as agências reguladoras da América Latina e Caribe mais confiam.

During the 1980s, the International Conference of Drug Regulatory Authorities and later the International Council for Harmonization of Technical Requirements for Pharmaceuticals for Human Use (ICH), launched in 1990, championed the idea of common technical and scientific standards toward pharmaceutical product authorization (1–4). Harmonization became a common practice among regulatory policymakers. However, implementing harmonized regulations at the country level proved to be a long and challenging endeavor (5). As a step forward, the concept of *reliance* has emerged as a new collaborative strategy among regulatory authorities (4, 6). Reliance is a broad concept that implies that a national authority uses the work done or the decisions taken by another trusted authority—usually a regulatory body from another jurisdiction—as an input for its regulatory procedures (4, 7). The direct recognition of marketing authorization (MA) from trusted authorities could be seen as the ultimate reliance (7).

During the last 15 years, several countries of Latin America and the Caribbean (LAC) have reformed their MA processes, aiming to directly recognize or abbreviate the MA of new chemical entities or biotherapeutic products earlier approved by the European Medicines Agency (EMA), the United States Food and Drug Administration (FDA), and a few others, including some regulators from among the countries of the Americas (8). In 2009, the Pan American Health Organization (PAHO) launched a qualification process intended to evaluate the regulatory authorities in the region and eventually recognize them as national regulatory authorities of reference (9). The evaluation system includes the requirement to rely on the regulatory decisions taken by a regulatory authority from another jurisdiction as part of the MA process at the national level (10).

The reliance of LAC regulators on MA decisions taken by regulatory authorities from other jurisdictions has seldomly been studied. Cañás et al. (11) have shown that up to 80% of the new drugs approved in Argentina in 2016 were first authorized by EMA or FDA following the recognition rule. In the meantime, concerns arose about MA flaws by stringent regulators (12).

In this study, we aimed to describe the current status of regulatory reliance in LAC by assessing the countries’ regulatory frameworks to approve new medicines, and to ascertain, for each LAC country, which foreign regulators are considered as trusted regulatory authorities to rely on.

## MATERIALS AND METHODS

We performed a cross-sectional study using official documents from the LAC regulatory authorities. All LAC countries were included. Official websites of the competent national regulatory authorities were systematically searched in order to identify the official regulation to approve new drugs.

A template for data extraction was developed. We documented information regarding the legal acceptance of any kind of direct recognition or abbreviated process for approval of new drugs at the country level, the exceptions to the mechanism, and whether the country accepts these rules regardless of the MA procedure followed by the foreign regulator (e.g., pathways for accelerated approval), the list of regulatory bodies recognized as trusted regulators to apply the rule, and name and year of the regulation. Data collection was carried out in December 2019 and completed in June 2020 for the Caribbean countries. The information was collected, analyzed, and summarized by two independent teams (authors CED and MC/MAU). Two consensus meetings were used to review the collected information and solve the observed discrepancies. These discrepancies varied in relevance. In three cases (Costa Rica, Honduras, and Mexico), the differences were related to the actual regulatory reliance status. For five countries (Ecuador, Guatemala, Panama, Paraguay, and Peru) the differences referred mainly to the identified trusted regulators and the existence of regulations other than those initially identified. Country experts from Honduras and Peru were contacted, as specific unclear information persisted after the consensus round.

We judged *direct recognition* or *abbreviated process* as when the country’s law explicitly mentioned one of these rules and the process was explained. The *direct recognition* of MA was defined as the approval of a new drug by a national regulator if the drug had been previously approved by a regulator from another jurisdiction. Direct recognition implies the omission of the revision of the clinical information dossier; however, other legal requirements at registration time could be kept, according to the country’s legislation. We defined *abbreviated process* as cases where the country regulator explicitly allows a fast-track registration for the drugs previously approved by another regulator. In such cases the clinical information dossier will be revised quickly.

Foreign regulatory bodies considered as *reference*—or trusted—regulatory authorities for MA reliance were collected as stated in the national legislation. If the regulation mentioned one or more European countries belonging to the European Economic Area, and therefore under the umbrella of the EMA’s central approval procedure for new drugs, we summarized them as EMA. If the regulation explicitly referred to PAHO’s list of national regulatory authorities of reference, we displayed them according to the information contained in the official PAHO webpage on the System for Evaluation of the National Regulatory Authorities of Medicines (13).

A descriptive analysis was performed to present the number of LAC countries accepting MA reliance as a formal regulatory practice, the number of foreign regulatory bodies that each country relies on, and the most-cited foreign regulators among the LAC regulatory frameworks.

## RESULTS

Regulatory documents regarding MA were found in 20 LAC regulators’ websites, covering 34 countries in the region. The 15 Member States of the Caribbean Community and Common Market (CARICOM) (Antigua and Barbuda, Bahamas, Barbados, Belize, Dominica, Grenada, Guyana, Haiti, Jamaica, Montserrat, Saint Kitts and Nevis, Saint Lucia, Saint Vincent and the Grenadines, Suriname, and Trinidad and Tobago) are signatories of a supranational regulatory unit, the Caribbean Regulatory System (CRS), and therefore were analyzed as a single regulatory framework in this study. After the consensus meetings and the advice from the country experts, the same 20 regulators were confirmed as the pool of regulators to be analyzed. Table 1 presents the included countries and the applicable regulations; these will be fully available by request.

Seven of the 20 included LAC regulators do not accept any form of MA reliance (Bolivia, Brazil, Chile, Cuba, Honduras, Nicaragua, and Venezuela). Thirteen LAC regulators either recognize or abbreviate the MA process in case of earlier approval by a regulatory authority from another jurisdiction (Argentina, Colombia, Costa Rica, Dominican Republic, Ecuador, El Salvador, Guatemala, Mexico, Panama, Paraguay, Peru, Uruguay, and the CRS). In 11 countries there are no exceptions to this mechanism. In Ecuador, the rule can only be applied if the active ingredient is already included in the national list of essential medicines, and never if the product was previously approved by a foreign regulatory authority under an accelerated (fast-track) procedure. The CRS registers a new drug when it has been approved by the designated trusted authority and only if the product is named in the most recent update of the WHO Model List of Essential Medicines or in the PAHO Strategic Fund list. The CRS recommends the reviewed drug to the Member States, and thereafter they have 60 days to confirm the MA.

**TABLE 1.** Applicable regulation to approve new medicinal products via regulatory reliance in Latin America and the Caribbean

N	Country	MA^*^ reliance Y/N	Name of the applicable regulation^**^	Regulation number^**^	Year
1	Argentina	Y	• Normas para el registro, elaboración, fraccionamiento, prescripción, expendio, comercialización, exportación e importación de medicamentos.	Decreto PEN N° 150/1992	1992
			• Resolución Conjunta del Ministerio de Economía y Finanzas Públicas y del Ministerio de Salud. Decreto N° 150/1992. Modificación.	Resolución Conjunta 452 y 1227	2014
2	Colombia	Y	• Reglamento parcial al régimen de registros y licencias, el control de calidad, así como el régimen de vigilancias sanitarias de medicamentos, cosméticos, preparaciones farmacéuticas a base de recursos naturales, productos de aseo, higiene y limpieza y otros productos de uso doméstico.	Decreto N° 677	1995
			• Decreto por el cual se establecen los requisitos y el procedimiento para las evaluaciones farmacológica y farmacéutica de los medicamentos biológicos en el trámite del registro sanitario.	Decreto 1782	2014
3	Costa Rica	Y	• Reconocimiento de la Evaluación y Aprobación de informes finales de Estudios Clínicos y no Clínicos por parte de las Autoridades Reguladoras de referencia como evidencia para el Registro Sanitario de Medicamentos.	Decreto Ejecutivo N° 39433-S	2015
4	Dominican Republic	Y	• Reglamento que regula la fabricación, elaboración, control de calidad, suministro, circulación, distribución, comercialización, información, publicidad, importación, almacenamiento, dispensación, evaluación, registro y donación de los medicamentos.	Decreto N° 246-06	2006
			• Resolución que establece los criterios para la aplicación del registro sanitario mediante procedimiento simplificado y el reconocimiento de los certificados de libre venta y certificados de buenas prácticas de establecimientos expedidos por las autoridades estrictas (OMS) y/o de las autoridades reguladoras de referencia regional (ARNr) de la Red PARF/OPS.	Resolución N° 000004	2016
			• Resolución que modifica los artículos segundo y tercero de la resolución No. 000004 de fecha 27 de Enero de 2016, que establece los criterios para la aplicación de registro sanitario mediante procedimiento simplificado y el reconocimiento de certificados de libre venta de productos y certificados de buenas prácticas de establecimientos, expedidos por las autoridades estrictas de la Organización Mundial de la Salud (OMS), autoridades reguladoras de referencia regional (ARNr) de la red PARF/OMS y por las autoridades de países de alta vigilancia.	Resolución N° 000021	2017
5	Ecuador	Y	• Reforma al reglamento sustitutivo de registro sanitario para medicamentos en general.	Acuerdo ministerial 2883	2013
			• Reforma al reglamento sustitutivo de registro sanitario para medicamentos en general.	Resolución ARCSA-DE-009-2018-JCGO	2018
			• Reforma y codificación del reglamento para la obtención del registro sanitario, control y vigilancia de medicamentos biológicos para uso y consumo humano.	Acuerdo ministerial 385-2019	2019
6	El Salvador	Y	• Reglamento especial para el reconocimiento de registros sanitarios extranjeros.	Decreto 34 / 2013	2013
7	Guatemala	Y	• Reconocimiento de registro sanitario de medicamentos aprobados por agencias regulatorias nivel IV según la OPS como base para tramitar homologación de registro en Guatemala.	Norma Técnica 077-2018	2018
			• Registro sanitario de referencia para productos biológicos y biotecnológicos.	Norma Técnica 67-2019	2019
8	Mexico	Y	• Acuerdo por el que se reconocen como equivalentes los requisitos establecidos en los artículos 167 y 170 del Reglamento de Insumos para la Salud y los procedimientos de evaluación técnica realizados por la Comisión Federal para la Protección contra Riesgos Sanitarios para el otorgamiento del registro sanitario de los insumos para la salud a que se refieren los artículos 2o. fracción XV inciso b y 166 fracción II del Reglamento de Insumos para la Salud, a los requisitos establecidos en la regulación 726/2004 de la legislación europea a fin de que la Comisión Europea autorice bajo el procedimiento centralizado la venta, distribución y uso de dichos insumos para la salud, en su territorio.	n/a	2012
			• Acuerdo por el que se reconocen como equivalentes los requisitos establecidos en los artículos 167 y 170 del Reglamento de Insumos para la Salud y los procedimientos de evaluación técnica realizados por la Comisión Federal para la Protección contra Riesgos Sanitarios para el otorgamiento del registro sanitario de los insumos para la salud a que se refieren los artículos 2o. fracción XV inciso b y 166 fracción II del Reglamento de Insumos para la Salud, a los requisitos solicitados, pruebas y procedimientos de evaluación realizados por la Administración de Alimentos y Medicamentos de los Estados Unidos de América para permitir en su país la venta, distribución y uso de dichos insumos para la salud.	n/a	2012
			• Acuerdo por el que se reconocen como equivalentes los requisitos establecidos en los artículos 167, 169, 170 y 177 del Reglamento de Insumos para la Salud y los procedimientos de evaluación técnica realizados por la Comisión Federal para la Protección contra Riesgos Sanitarios para el otorgamiento del registro sanitario de los insumos para la salud a que se refieren los artículos 2o., fracción XV, inciso b y 166, fracción II del Reglamento de Insumos para la Salud, con relación a los artículos 222 Bis y 229 de la Ley General de Salud, a los requisitos solicitados, pruebas y procedimientos de evaluación realizados por la Agencia Suiza para Productos Terapéuticos-Swissmedic para permitir en su país la venta, distribución y uso de dichos insumos para la salud.	n/a	2012
			• Acuerdo por el que se reconocen como equivalentes los requisitos establecidos en los artículos 167 y 170 del Reglamento de Insumos para la Salud y los procedimientos de evaluación técnica realizados por la Comisión Federal para la Protección contra Riesgos Sanitarios para el otorgamiento del registro sanitario de los insumos para la salud a que se refieren los artículos 2o. fracción XV inciso b y 166 fracción II del Reglamento de Insumos para la Salud, a los requisitos solicitados, pruebas y procedimientos de evaluación realizados por la Administración de Productos Terapéuticos de Australia para permitir en su país la venta, distribución y uso de dichos insumos para la salud.	n/a	2012
			• Acuerdo por el que se reconocen como equivalentes los requisitos establecidos en los artículos 167, 169, 170 y 177 del Reglamento de Insumos para la Salud y los procedimientos de evaluación técnica realizados por la Comisión Federal para la Protección contra Riesgos Sanitarios para el otorgamiento del registro sanitario de los insumos para la salud a que se refieren los artículos 2o., fracción XV, inciso b y 166, fracción II del Reglamento de Insumos para la Salud, con relación a los artículos 222 Bis y 229 de la Ley General de Salud, a los requisitos solicitados, pruebas y procedimientos de evaluación realizados por el Ministerio de Salud de Canadá para permitir en su país la venta, distribución y uso de dichos insumos para la salud.	n/a	2012
9	Panama	Y	• Procedimiento abreviado para el registro sanitario de medicamentos, su renovación y modificaciones.	Decreto Ejecutivo N°58	2017
			• Ley 97 que modifica y adiciona artículos a la Ley 1 de 2001, sobre medicamentos y otros productos para la salud humana, y dicta otras disposiciones.	Ley 97	2019
10	Paraguay	Y	• Ley de productos para la salud y otros.	Ley N° 1119	1997
			• Ley de protección de información no divulgada y datos de prueba para los registros farmacéuticos.	Ley N° 3283	2007
			• Reglamento al artículo 24 de la Ley N° 1119/1997, "De productos para la salud y otros", y se establecen los requisitos para el registro de medicamentos biológicos.	Decreto N° 6611	2016
11	Peru	Y	• Ley de los productos farmacéuticos, dispositivos médicos y productos sanitarios.	Ley N° 29459	2009
			• Reglamento para el registro, control y vigilancia sanitaria de productos farmacéuticos, dispositivos médicos y productos sanitarios.	Decreto Supremo N° 016-2011-SA	2011
			• Modificación del reglamento para el registro, control y vigilancia sanitaria de productos farmacéuticos, dispositivos médicos y productos sanitarios y el reglamento de establecimientos farmacéuticos a efecto de incluir a Austria como país de alta vigilancia sanitaria.	Decreto Supremo N° 018-2019-SA	2019
12	Uruguay	Y	• Modificación del art. 7 del Decreto 324/999 relativo a medicamentos y productos afines de uso humano.	Decreto N° 28/014	2014
			• Registro Sanitario de Medicamentos Biológicos.	Decreto N° 38/015	2015
13	Caribbean Regulatory System^***^	Y	• Caribbean Pharmaceutical Policy.	n/a	2013
14	Bolivia	N	• Ley del Medicamento.	Ley N° 1737	1996
			• Reglamento a la Ley del Medicamento.	Decreto supremo 25235	1998
15	Brazil	N	• Resolução da Diretoria Colegiada - RDC N° 200, de 26 de Dezembro de 2017.	RDC N° 200	2017
			• Dispõe sobre o registro de produtos biológicos novos e produtos biológicos e dá outras providências.	RDC N° 55	2010
16	Cuba	N	• Reglamento para el registro sanitario de medicamentos de uso humano.	Resolución Ministerial N° 321	2009
17	Chile	N	• Reglamento del Sistema Nacional de Control de los Productos Farmacéuticos de Uso Humano.	Decreto Supremo 3/10	2011
18	Honduras	N	• Reglamento para el control sanitario de productos, servicios y establecimientos de interés sanitario.	Acuerdo 06	2005
19	Nicaragua	N	• Ley de Medicamentos y Farmacias.	Ley 292	1998
20	Venezuela	N	• Ley de Medicamentos.	n/a	2000
			• Norma para el registro, liberación de lotes y control de los productos biológicos.	N-PERC-001	2008

* MA: Marketing authorization

** Names and numbers of the applicable regulation are expressed in the official language of the country.

*** The Caribbean Regulatory System is a collaborative initiative acting as a regulatory unit for 15 Member States of the Caribbean Community and Common Market – CARICOM (Antigua and Barbuda, Bahamas, Barbados, Belize, Dominica, Grenada, Guyana, Haiti, Jamaica, Montserrat, Saint Kitts and Nevis, Saint Lucia, Saint Vincent and the Grenadines, Suriname, Trinidad and Tobago).

***Source:*** Prepared by the authors from the study data.

**FIGURE 1.**
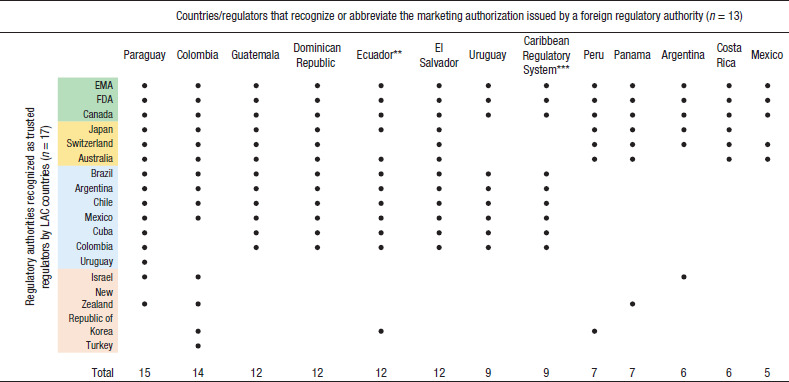
Reference (trusted) regulatory authorities for Latin American and Caribbean countries^*^

***Notes***: EMA, European Medicines Agency; FDA, United States Food and Drug Administration

Figure 1 lists the LAC countries and the corresponding foreign regulators on which they rely. Paraguay (*n* = 15) and Colombia (*n* = 14) are the countries that rely on the highest number of foreign regulatory authorities. Paraguayan regulation explicitly allows reliance on MA issued by any of the Member States of the Southern Common Market (MERCOSUR by its abbreviation in Spanish), PAHO’s national regulatory authorities of reference, EMA, and some others. Colombia is the only LAC country abbreviating the approval pathway in case of earlier approval by any of 36 Member States of the Organisation for Economic Co-operation and Development (OECD). A second group of six regulators (Dominican Republic, Ecuador, El Salvador, Guatemala, Uruguay, and the CRS) all rely on EMA, FDA, and Health Canada’s MA, as well as on the authorizations issued by any of PAHO’s national regulatory authorities of reference. Finally, a third group of five countries (Argentina, Costa Rica, Mexico, Panama, and Peru) rely on regulatory authorities from Europe, the United States of America, Canada, Oceania, and Asia, but not on any LAC regulator.

EMA, FDA, and Health Canada are the trusted regulatory authorities for all 13 of the LAC regulators included. Ten of them also rely on the regulatory decisions taken by Australia, Japan, and Switzerland. Argentina, Brazil, Chile, and Mexico are reference authorities for eight countries. Colombia and Cuba are trusted parties for seven regulators, and finally, Israel, Republic of Korea, New Zealand, Turkey, and Uruguay are each cited as trusted regulatory authorities in one to three countries’ regulatory frameworks (see Figure 1).

## DISCUSSION

In this study, we systematically reviewed and documented the current state of regulatory reliance for the approval of new medicinal products in the 34 countries of LAC. To the best of our knowledge, this is the first study exploring the normative status allowing MA reliance in the region. Our results show that LAC countries have adopted the concept of MA reliance, particularly in the last 10 years. Indeed, 65% of LAC regulators (out of 20) are currently relying on other regulators’ decisions. In other words, decisions around MA taken mainly by EMA, FDA, or Health Canada are fully recognized by two-thirds of regulators in the LAC region.

Most regulatory authorities in the region have taken this decision based on the idea of having limited resources to deal with a complete and critical appraisal, particularly of the new biotechnological drugs. In 2016, Ahonkhai et al. (14) observed a time lapse of one to two years for pharmaceuticals registration in sub-Saharan Africa after registration by stringent regulators. To reduce the registration time of medicines and vaccines in low- and middle-income countries, the authors proposed relying on stringent agencies as a strategy to optimize the MA system. Similarly, a group of regulatory experts and researchers have recently outlined recommendations on financial priority setting to strengthen regulatory systems globally (6). The first recommendation was to advance and leverage convergence and reliance initiatives as part of the implementation of value-added regulatory practices to increase timely access to quality-assured medical products. Based on a similar rationale, regulatory reliance has been supported by the pharmaceutical industry (15), the International Conference of Drug Regulatory Authorities (16), and the World Health Assembly (17).

However, the practice of fully relying on regulatory authorities from another jurisdiction to approve new drugs engenders at least three potential problems. First and most important is the growing evidence about flaws in the EMA and FDA’s appraising of the supporting evidence to approve new drugs. For instance, up to 49% of the clinical trials underpinning the authorization of new cancer drugs by EMA scored as having a high risk of bias when those were independently evaluated (18). EMA as well as FDA have been approving new cancer drugs based on surrogate outcomes (19), and mostly in non-randomized clinical trials (20, 21). Whenever the same surrogate outcomes were measured in randomized trials, the duration of response was lower in 87% of the studied indications (22). Moreover, disappointing results have emerged when strong outcomes—i.e., overall survival or quality of life—were measured as primary end-points in the clinical trials (21, 23).

Second, both EMA and FDA have implemented specific programs to abbreviate the approval process to obtain quicker access to certain new drugs. Under these programs, selected new drugs, mostly biotechnological products, are approved on the basis of non-randomized trials, surrogate outcomes, and usually on phase II of the clinical development of the drug (23–25), with the consequences mentioned in the previous paragraph. Twelve LAC regulators easily provide an MA whenever a drug is approved under an accelerated procedure by a trusted party, and maintain this without further evaluation. Only one country (Ecuador) adapted the legislation to ban the direct recognition of new drugs if these were approved under a fast-track mechanism. This could be an example of local adaptation of the regulatory framework.

* Bolivia, Brazil, Chile, Cuba, Honduras, Nicaragua, and Venezuela do not accept any form of MA reliance.

** Ecuadorian legislation does not accept reliance on a marketing authorization if the drug is not included in the national list of essential medicines and if it has been approved under a fast-track process in the trusted regulator.

*** The Caribbean Regulatory System (CRS) is a collaborative initiative acting as regulatory unit for 15 Member States of the Caribbean Community and Common Market – CARICOM (Antigua and Barbuda, Bahamas, Barbados, Belize, Dominica, Grenada, Guyana, Haiti, Jamaica, Montserrat, Saint Kitts and Nevis, Saint Lucia, Saint Vincent and the Grenadines, Suriname, and Trinidad and Tobago). The CRS registers a drug if has been approved by the designated trusted authority, and only if the product is named in the most recent update of the WHO Model List of Essential Medicines or in the PAHO Strategic Fund list.

***Source***: Prepared by the authors from the study data.

Third, when an LAC regulator relies on a national regulatory authority of reference under the PAHO qualification process (13) a chain of *secondary reliance* can occur. This could happen when one of the LAC regulators assessed by PAHO has approved a given drug by relying on a trusted party, and then one of the LAC non-trusted regulators decides to approve the same drug by relying on that particular PAHO reference authority. This is not easy to detect on regulators’ databases, given the difficulties in identifying which products were approved based on reliance mechanisms and which ones were not.

Regarding the PAHO system of national regulatory authorities of reference, the idea of granting *reference* regulators was brought by Argentina, Brazil, Chile, Colombia, Cuba, and Mexico (together known as the Oaxaca Group). The qualification process to become a *reference authority* was launched in 2009 (13). To date, regulators from Argentina, Brazil, Canada, Chile, Colombia, Cuba, Mexico, and the United States of America have been recognized as such (13). These eight reference regulators have become trusted authorities for seven LAC regulators (Dominican Republic, Ecuador, El Salvador, Guatemala, Paraguay, Uruguay, and the CRS, representing 15 countries)**.** Hence, the qualification process by PAHO has important implications on the regulatory processes in the region. One would expect that countries with lower regulatory capacity would use reliance mechanisms more than countries with high capacity. However, there is no apparent pattern linking the maturity level of the authority to the extent of reliance, or the countries of reference. For example, while Colombia is a PAHO reference authority, it relies on an larger number of countries than other LAC countries; whereas Costa Rica, being a mid-level regulator according to the PAHO system, relies on fewer countries. Furthermore, it must be noted that none of the LAC regulators recognized as PAHO reference bodies are actually mutually trusting on each other’s MA decisions.

Paraguay and Colombia must be highlighted as the countries with the higher numbers of trusted regulators to rely on. In the case of Paraguay, it is because the regulatory framework explicitly allows reliance on the MERCOSUR countries, besides PAHO’s reference authorities, the EMA, and some other international regulators. In the case of Colombia, the regulation states that it is possible to rely on any of the 36 OECD countries, which officially joined in April 2020. Finally, the CRS represents a unique and novel initiative in the region, as it aggregates specific regulatory duties for the 15 CARICOM Member States, including MA revision and registration when a given drug has been previously approved by a trusted party and listed in the WHO Model List of Essential Medicines or in the PAHO Strategic Fund list (26).

In relation to the countries that do not include MA reliance in their regulatory legal framework, Bolivia, Cuba, Nicaragua, and Venezuela initiated in 2009, together with some others, the project of a supranational regulatory agency for the Member States of the Bolivarian Alliance for the Peoples of Our America (ALBA by its Spanish abbreviation) (27). It is possible that the decision not to adhere to a broad international reliance was aimed at strengthening the ALBA supranational regulator. Brazil is the second largest emerging pharmaceutical market in the world (28). The country’s agency (ANVISA by its Portuguese abbreviation) was created in 1999 (29) and accepted as a member of the ICH in 2016 (28). ANVISA has received continuous political support to upraise the country’s regulatory capacities, including those that deal with their own appraisal of new drugs. A similar policy could be seen in the Institute of Public Health, the Chilean regulator (30).

Reliance may be a double-edged sword. While optimizing MA to speed access to essential medicines may be desirable, regulatory efficiency must not jeopardize in-depth and careful evaluation of new medicines. Reliance may foster cooperation and resource optimization, but it may also decrease transparency of approval pathways, induce loss of national control over approvals of doubtful new medicines, and promote quick approvals where capacities to supervise them after MA, and even to cancel the MA, is almost nonexistent.

A potential limitation of our study is that most countries which have reformed their legal frameworks to allow reliance did this only in recent years and, therefore, the implementation could differ greatly among them. We did not evaluate the level of implementation of such policies at country level; it could be the case that a changed legal framework had not yet been implemented, either partly or completely, due to particular local reasons. This aspect deserves more attention in future research.

### Conclusion

Regulatory reliance has become a common regulatory practice among LAC regulatory authorities. Thirteen out of 20 regulators, covering 34 countries, directly recognize or abbreviate the MA process in case of earlier approval by a regulator from another jurisdiction. The regulatory authorities that are most relied upon are EMA, FDA, and Health Canada.

Reliance might be a good regulatory practice if i) decisions taken by trusted regulators are based on the highest quality evidence and critically appraised, ii) decisions are adapted to the local context, and iii) strong teams exist within the national regulatory authorities to deal with new contrasting evidence that could emerge and change the initial appraisal of a drug. Unless these conditions are met, reliance can also lead to indiscriminate approval of products with unknown benefit.

## Disclaimer.

Authors hold sole responsibility for the views expressed in the manuscript, which may not necessarily reflect the opinion or policy of the *RPSP/PAJPH* and/or PAHO.
